# Why medical students choose not to carry out an intercalated BSc: a questionnaire study

**DOI:** 10.1186/1472-6920-10-25

**Published:** 2010-03-23

**Authors:** Jamie A Nicholson, Jennifer Cleland, John Lemon, Helen F Galley

**Affiliations:** 1 Division of Applied Medicine, School of Medicine & Dentistry, University of Aberdeen, Aberdeen, AB25 2ZD, UK; 2 Division of Medical & Dental Education, School of Medicine & Dentistry, University of Aberdeen, Aberdeen, AB25 2ZD, UK; 3 Directorate of Information Technology, Edward Wright Building, University of Aberdeen, Aberdeen, AB24 3QY, UK

## Abstract

**Background:**

At some medical schools, students can opt to undertake a 1 year intercalated degree, usually a BSc, in addition to their medical course. Over the last few years the numbers of students who have opted to undertake an intercalated degree have been steadily decreasing despite the advantages in securing foundation posts. The aim of this study was to find out why medical students opted not to take an intercalated degree.

**Methods:**

All 4^th ^and 5^th ^year medical students (n = 343) who had elected not to take an intercalated degree were personally handed a questionnaire.

**Results:**

293 completed questionnaires were returned (response rate 85%). The most common reason students opted not to intercalate was because they did not want to have another year of study (69.6%) or incur more debt (51.9%). Only 45 (15.3%) students said they had enough information to inform their decision: reported take up of information provision was poor.

**Conclusions:**

Our findings indicate that the benefits of intercalating need to be better defined and presented to students in a way that they can make a more informed decision.

## Background

Approximately a third of UK medical students undertake a one year intercalated degree, usually a BSc, in addition to their MBChB degree. In most medical schools this is optional (e.g. Kings College London, Aberdeen, Leeds) but in others the extra year is compulsory (e.g. Imperial College and University College, London) [1,2]. At the University of Aberdeen the numbers of students undertaking the intercalated BSc has been declining; over the last four years only 17.9% chose to do an intercalated degree; in 2007/8 only 24 students out of 185 (12.9%) intercalated.

Competition for doctors' jobs is a subject of which few in the profession are unaware [3]. In a survey of pre-registration house officers (PRHOs) by the British Medical Association in 2005, 10% of the 680 PRHOs who replied were not working as doctors [4]. Final year students have also expressed concerns about securing their first job [5]. The PRHO scheme was superseded by foundation training in 2005. The UK foundation programme awards an extra 6 points for a first class BSc in addition to a medical degree, the same as a PhD, and prizes, publications and research presentations accord 4 more points. Therefore an extra 10 points are potentially available for students with an intercalated BSc. The total number of points available is 100 (40 for medical school academic ranking and 60 for application questions) and it may be perceived that an extra 10 points is of little significance. However, this is not the case, since only 6 points separates the highest academic ranking students from the lowest, and of course transferable skills gained during an intercalated degree, though less tangible, will contribute to the answers to the application questions [6]. We have also shown that at the University of Aberdeen, intercalating also adds benefits in terms of subsequent improved performance upon return to the MBChB, thus further increasing the academic ranking for foundation posts [7].

In 2004 there were 500 fewer clinical academics employed in UK medical and dental schools than in 2001 [2,8]. Graduates who have taken an intercalated degree are more likely to have a career in academic medicine [9,10] and intercalated BSc graduates are more likely to practice evidence based medicine even if they do not go into academic posts [11].

The numbers intercalating varies between medical schools. In a survey of second year medical students at a London medical school 84.5% answered 'yes' to the question "Do you want to do an intercalated degree?" although only around 50% of the students actually went on to undertake an intercalated degree [12]. At the University of Leeds around half the students voluntarily undertake intercalated degrees and numbers have been rising year by year (A. Hay, personal communication). However, most students in our university actively choose not to undertake an intercalated degree. This seems puzzling given the potential gains of doing so e.g. potential for publications, prizes and long term career progression. We therefore conducted a questionnaire study to find out the reasons why students made this choice.

## Methods

At the University of Aberdeen undergraduate medical students can undertake an intercalated BSc (Hons) degree in Medical Sciences. Our programme lasts for one year and students usually commence the programme after year 3 but may also undertake the intercalated degree after year 4. As the course is a level 4 course, students must have already completed at least 3 years of the MBChB. The degree programme consists of four interlocking taught courses specifically tailored to medical students, providing structured teaching over a 12 week period and building up to a detailed self-directed group project. This is followed by an extended individual 20 week research project, with projects available in a large range of departments and disciplines.

We were advised by the chair of the North of Scotland Research Ethics Committee that approval was not required. However, students were made aware that the questionnaire was completely anonymous and participation was entirely voluntary. The questionnaire (see Additional file 1) was initially piloted on a small group of students to ensure clarity and lack of ambiguity. It was then handed personally to all 4^th ^and 5^th ^year medical students who had opted not to do an intercalated degree. Those who had completed an intercalated degree were purposely excluded; however all other students were included, even though some may have already undertaken a previous degree before studying medicine.

The first few questions addressed demographic information including age, ethnic origin, sex and education. We asked students to choose the main reason why they did not want to do an intercalated degree from a set of statements. A similar structure was used to find out what benefits students thought were associated with an intercalated degree. We asked students closed questions about debt levels and funding, questions about any interest in research and what their career ambitions were. There were also questions about the adequacy of the information provided about the intercalated BSc. Finally students were asked if they regretted their decision and any comments were invited.

SNAP survey software was used to capture the data which was analysed using SPSS Version 15.0 for Windows.

## Results

343 medical students, of whom 164 were entering 4^th ^year and 179 were entering 5^th ^year, did not undertake an intercalated degree when it was offered in 2006 or 2007 (Table 1). Of these, 293 (137 from 4^th ^year and 156 from 5^th ^year) students completed the questionnaire, giving a response rate of 85%.

**Table 1:** Response rate and male/female distribution

Cohort	Number invited	Number (%) responded	Number (%) males	Number (%) females
**Total**	343	293 (85.4)	108 (36.9)	185 (63.1)
**4^th ^year**	164	137 (83.5)	51 (37.1)	86 (62.9)
**5^th ^year**	179	156 (87.2)	57 (36.5)	99 (63.5)

The majority who completed the questionnaire were female (172, 63.0%). There was a small percentage of postgraduates (67, 22.9%) who already had a BSc or higher degree, 76.8% were white (British, Irish or other). The age ranges of those who responded are shown in Table 2. In the cohort of 5^th ^year students there were no students under 20 years and all but one was in the age range 21-24 years.

**Table 2:** Age distribution of responders

Cohort	Number (%) <20 Years	Number (%) 21-22 years	Number (%) 23-24 years	Number (%) 25-30 years	Number (%) >31 years
**Total**	34 (12.4)	141 (51.5)	87 (31.7)	8 (2.9)	4 (1.5)
**4^th ^year**	34 (26.2)	72 (55.4)	13 (10)	8 (6.2)	3 (2.3)
**5^th ^year**	0	69 (47.9)	74 (51.4)	0	1 (0.7)

**Figure 1:**
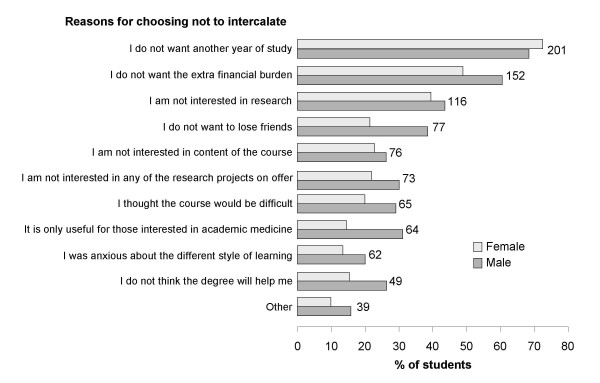
**Males and female student choosing each reason for deciding they did not wish to undertake an intercalated degree**.

Sixty seven (22.9%) students already had a first degree and this was the primary reason why they did not opt to undertake an intercalated degree. Of the remaining 226 students, not wanting an extra year of study was the most common reason for not doing an intercalated degree (201, 88.9%) and not wanting any extra financial burden was the next most common (152, 67.3%) (Figure 1). The percentage of students choosing each reason was similar for males and females, except that more males than females indicated the degree was only useful for those interested in academic medicine (31.7% of males *vs*. 15.7% of females, p = 0.003, Continuity Correction Test), and that they thought the degree would not help them (30.7% *vs*. 16.3%, p = 0.008, Continuity Correction) (Figure 1). Younger students were more likely to have expressed concern about the difficulty of the intercalated degree course (Likelihood ratio, p = 0.033).

Students were asked to indicate their debt within specified ranges (Table 3). Only 69 students (25.7%) had no debt. More than half had debts of over £5,000, and nearly a third had debts over £15,000 (75, 27.9%). Of the 41 students who indicated that they had no debt, 28 (40.6%) indicated that financial restraint was their main reason for not intercalating, and of the 112 students with debt over £10,000, 68 (60.7%) also gave this reason. More of the students in 5^th ^year had debt over £10K compared to students in 4^th ^year (54.1% vs. 28.3%, Fischer's Exact Test, p < 0.0001, Table 3).

**Table 3:** Student debt

Cohort	Number (%) No debt	Number (%) <£5,000	Number (%) £5,000-£10,000	Number (%) £10,001-£15,000	Number (%) £15,001-£20,000	Number (%) £20,001-£25,000	Number (%) >£25,001	Number (%) >£10,000
**Total**	69 (25.7)	37 (13.8)	51 (19.0)	37 (13.8)	45 (16.7)	15 (5.6)	15 (5.6)	112 (41.6)
**4^th ^year**	38 (29.2)	20 (15.4)	35 (26.9)	18 (13.8)	15 (11.5)	2 (1.5)	2 (1.5)	37 (28.5)
**5^th ^year**	31 (22.3)	17 (12.2)	16 (11.5)	19 (13.7)	30 (21.6)	13 (9.4)	13 (9.4)	75* (54.0)

* Significantly higher than in 4^th ^year cohort (p < 0.0001, Fischer's Exact test).

**Figure 2:**
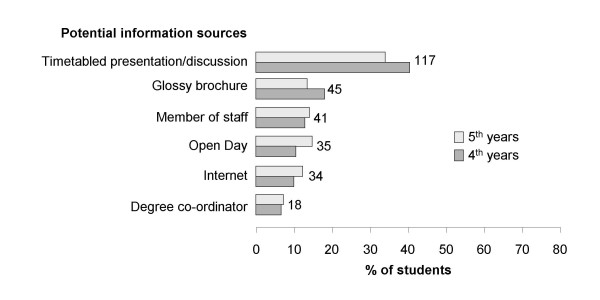
**4^th ^and 5^th ^year students who indicated the information sources they had used to inform their decision as to whether or not they undertook an intercalated degree**.

Information about intercalating is provided to 3^rd ^year students in the form of a timetabled presentation in October followed by a question and answer session with previous intercalated BSc graduates. An information brochure is also available and an Open Day, when poster displays of available research projects are provided and potential research supervisors and BSc degree staff are available to answer questions, takes place in November. In our questionnaire students were asked if they felt they had been provided with enough information to inform their decision; 84.6% (248) said they had not. However only 39.9% (117) of students said they went to the presentation, only 15.4% (45) read the brochure and only 11.9% (35) went to the Open Day (Figure 2). Uptake of these information sources was similar for both cohorts of students (Figure 2).

**Figure 3:**
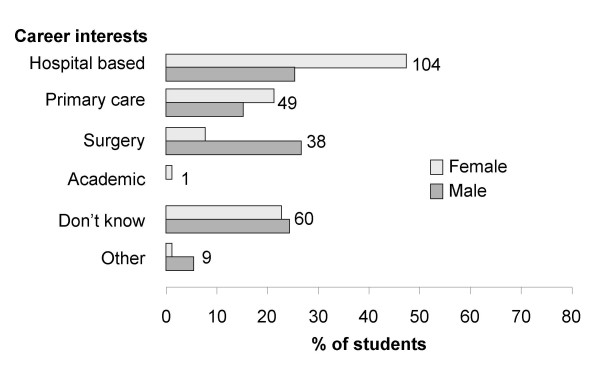
**Male and female students who indicated their future career interests**.

Figure 3 shows that most students were aiming for a hospital based career (104, 39.8%), followed by primary care/general practice (49, 18.8%) and surgery (38, 14.6%). Sixty students (23.0%) said they did not know what their career path would be. Only one student indicated an academic career as their preference. Men were almost four times more likely to choose surgery than women (27.4% *vs*. 7.2%, p = 0.007 Linear-by-Linear Association). Almost half the students (135, 49.3%) said they were not interested in research; only 16.4% (45) said they were. Of those who were interested in research, 41% (17) already had a degree and this group were 3.5 times more likely to say they had an interest in research compared to students without a degree.

Around a quarter of the students (70, 23.8%) indicated that they might have been interested in an intercalated degree in another topic. Suggestions for potential subjects areas were invited and included anatomy and physiology. In addition, there was some interest for non-science topics such as arts and languages.

Students were also asked to indicate from a list, what they thought were the benefits of an intercalated degree; 224 (76.5%) agreed that it would provide experience in research; 188 (64.2%) agreed that new skills would be learned and 166 (56.7%) thought an intercalated BSc would broaden their knowledge. Just under half (145, 49.5%) thought it would improve long term career prospects and 122 (41.6%) thought it would help then get a job in the future. When asked, 50 (18.7%) students indicated that they now regretted their decision not to undertake an intercalated BSc and this entire group of respondents expressed concerns over job competition.

## Discussion

In our questionnaire survey we found that the most common reason students gave for not intercalating was not wanting another year of study. The second most common reason was not wanting further financial burden as a result of an extra year of study. Despite this, most of the students recognised the benefits associated with an intercalated degree.

We were surprised that students, although they recognised the benefits of having an additional qualification and the associated experience and transferable skills, simply could not see beyond the extra year of study. We were also disappointed that students felt they did not get enough information about the degree, although a large number of students did not make use of the information sources which were available.

Most students had debt in the £5,000-£10,000 bracket; only 26% had no debt at all, despite the second most popular reason against intercalating being avoidance of financial burden. However the debt range did not correlate with choosing financial burden as a reason against intercalating and the debt of students who gave finances as a reason not to intercalate and those who did not, were similar. As might be expected more students in 5^th ^year had accrued debt over £10,000, although the percentage of students indicating financial reasons for not intercalating was similar (52.3% of 4^th ^year students and 51.3% of 5^th ^year students).

A previous study at this University examined medical students' debt levels in relation to performance in examinations [13]. Little relationship was found between actual debt and performance, although students' perception of debt did relate to performance. It is possible that perceived rather than actual debt may have resulted in our students not undertaking an intercalated degree although it is clear that many students did have considerable debt.

The decline in academic medicine in the UK is now widely recognised [2,8]. Our study showed that those students who were interested in research were more likely to have a previous degree, in agreement with a study some 10 years ago [1]. This may suggest that if medical students had some exposure to research prior to making the decision about intercalating, an interest in research might be kindled [14]. With the competition for some popular specialities [15], doctors may have to consider other choices. Options such as MB PhD courses can offer an alternative path to an academic/research career [16] although these have been slow in gaining popularity in the UK.

The merit of 'non science' based degrees in terms of a student's medical career is uncertain. In the foundation application scoring system, similar points are awarded for BA degrees as for BSc, MSc or PhD, but fewer points for BMedSci degrees, notably those degrees that do not involve an extra year of study. Our university is now offering more choice and students can now undertake an BSc in Medical Humanities or join a selection of Honours year courses of existing science degrees including anatomy, sports science and neuroscience in addition to the BSc in Medical Science. Such an increase in student choice may lead to more students intercalating.

An intercalated degree gives students early exposure to research and has been shown to help develop valuable skills for life long practice. Much work needs to be done to address the negative views held by students and the benefits of intercalating need to be better defined and presented to students in such a way that they can make an informed decision that maximises their future potential. Much of the concern of a few years ago about jobs for newly qualified doctors was in the event unfounded, and indeed 100% of UK applicants were allocated to foundation schools across the UK in 2007, 2008 and 2009 [6]. However, the competition for foundation training posts in specific foundation schools is fierce, and places for academic training programmes in particular, are oversubscribed. It will be interesting to see if the competition for intercalated degree places also increases.

This study had an excellent response rate and was thus highly representative of the entire group who did not intercalate. This is a major strength of the study and was achieved by physically handing questionnaires to each individual student as they attended for registration. Our study was larger than any other published single site survey (almost 300 students). However, our study was limited in that it was at a single site, although the systems for intercalating at other medical schools do vary considerably between universities such that comparisons are difficult to interpret. A repeat of this survey in a few years' time will enable us to see if the wider choice of intercalated degrees affects the numbers of student who make the decision not to intercalate.

## Conclusions

We found that despite recognising the benefits associated with an intercalated degree many students choose not to intercalate at our university. The most common reason students gave for not intercalating was not wanting another year of study. This may be related to insufficient student choice.

## Competing interests

The authors declare that they have no competing interests.

## Authors' contributions

JAN participated in designing the questionnaire, analysed the data and prepared the figures and edited the paper. HFG instigated and conceived of the study, co-ordinated data collection, participated in designing the questionnaire and drafted the manuscript. JC participated in designing the questionnaire and editing the manuscript. JL edited the questionnaire and captured the data. All authors read and approved the final version.

## Pre-publication history

The pre-publication history for this paper can be accessed here:

http://www.biomedcentral.com/1472-6920/10/25/prepub

## Supplementary Material

Additional file 1 **Questionnaire**. The is the questionnaire given to students. Click here for file
