# Supplementary material for: Why medical students choose not to carry out an intercalated BSc: a questionnaire study
Source: BMC Med Educ. 2010 Mar 23;10:25. doi: 10.1186/1472-6920-10-25 (PMC2850914; doi:10.1186/1472-6920-10-25)
Supplement: Additional file 1 — Questionnaire. The is the questionnaire given to students. [file 1472-6920-10-25-S1.PDF]

UNIVERSITY OF ABERDEEN

Intercalated BSc

This questionnaire is ANONYMOUS. Please do not write your name or ID number on it!

Please complete by ticking the relevant box(es)

Q1 Are you

Male .... ☐ *Female* .... ☐

Q2 How old are you?

19-20 .... ☐ *21-22* .... ☐ *23-24* .... ☐ *25-30* .... ☐ *31-35* .... ☐ *35+*........... ☐

Q3 Please tell us if you are:

- *White* ☐
- *White - British* ☐
- *White - Irish* ☐
- *Other- White background* ☐
- *Black or Black British -Caribbean* ☐
- *Black or Black British - African* ☐
- *Other Black background* ☐
- *Asian or Asian British - Indian* ☐
- *Asian or Asian British - Pakistani* ☐
- *Asian or Asian British - Bangladeshi* ☐
- *Chinese or Other Ethnic background - Chinese* ☐
- *Other Asian background* ☐
- *Mixed - White and Black Caribbean* ☐
- *Mixed - White and Black African* ☐
- *Mixed - White and Asian* ☐
- *Other Mixed background* ☐
- *Other Ethnic background* ☐
- *Not known* ☐

Q4 Please tell us about your previous education (Tick all those that apply):

- *Scottish Higher/National Qualification* ☐
- *A Level* ☐
- *Irish Leaving Certificate* ☐
- *International Baccalaureate* ☐
- *Diploma e.g. ONC/OND/HNC/HND* ☐
- *BSc* ☐
- *MSc* ☐
- *MPhil* ☐
- *PhD* ☐
- *Other* ☐

Q5 Did you initially apply to do the Intercalated BSc ?

Yes .... ☐ Go to Q6 *No* .... ☐ Go to Q10

Q6 Were you offered a place?

Yes .... ☐ Go to Q7 *No* .... ☐ Go to Q10

Q7 Which of your project choices were you offered?

1st................... ☐ *2nd*.................. ☐ *3rd* .... ☐ *4th*................... ☐ *Other*............... ☐

Q8 Did you accept the offer given to you ?

Yes .... ☐ Go to Q11 *No* .... ☐ Go to Q9

Q9 Why did you withdraw your application ?

- *Because of failure to get 1st choice of project* ☐
- *Because of failure to get 2nd choice of project* ☐
- *Because of failure to get 3rd choice of project* ☐
- *Because of failure to get 4th choice of project* ☐
- *Because of funding concerns* ☐
- *Other* ☐

Q10 Do you intend to apply for the BSc in 4th year instead?

- *Yes* ☐
- *No* ☐
- *Have been offered a place for after 4th year already* ☐

Q11 What career do you have in mind ?

- *Academic* ☐
- *Primary Care/GP* ☐
- *Hospital Based* ☐
- *Surgery* ☐
- *Don't know* ☐
- *Other* ☐

Q12 Are you interested in research ?

- *Yes* ☐
- *No* ☐
- *Don't know* ☐
- *Possibly* ☐

Q13 How are you funding your medical degree ? (Tick all those that apply)

- *Loan* ☐
- *Self support* ☐
- *Parental support* ☐
- *Bursary* ☐
- *Sponsorship* ☐
- *Other* ☐

Q14 How much debt have you accumulated so far?

- *No debt* ☐
- *0-£5,000* ☐
- *£5,000-£10,000* ☐
- *£10,000-£15,000* ☐
- *£15,000-£20,000* ☐
- *£20,000-£25,000* ☐
- *£25,000+* ☐

Q15 Please tell us whether you did any of the following (Tick all those that apply)

- *Attended the presentation on the BSc* ☐
- *Went to the open day on the BSc* ☐
- *Read the Aberdeen BSc brochure* ☐
- *Conducted any background reading, internet search, etc. on the BSc degree* ☐
- *Spoke to the BSc co-ordinator (Dr Helen Galley) about the degree* ☐
- *Spoke to another member of staff about the degree* ☐
- *Had adequate information and guidance in deciding on whether to do the Intercalated BSc* ☐

Q16 Would you have been interested in carrying out an Intercalated BSc at another university ?

- *Yes* ☐
- *No* ☐
- *Maybe* ☐

Q17 Would you have liked to study an Intercalated BSc in another subject ?

- *Yes* ☐
- *No* ☐

If Yes what subject would you have liked to study ?

Q18 Did you speak to the BSc co-ordinator (Dr Helen Galley) about it ?

- *Yes* ☐
- *No* ☐

Designed by SNAP software

Q19 Have the changes to MMC (Modernising Medical Careers) and doctor training, made you rethink your decision to take up the Intercalated BSc ?

Yes ☐ *No* ☐

If Yes what made you re-think your decision ?

Q20 The following is a list of reasons why you might have chosen not to do an Intercalated BSc. Please tick ONE box in the relevant column to indicate the MAIN reason you chose not to do a BSc and tick as many boxes as you like in the OTHER column.

	Main	*Other*
I do not want another year of study	☐	☐
I do not want the extra financial burden	☐	☐
It is only useful for those interested in academic medicine	☐	☐
I am not interested in research	☐	☐
I am not interested in content of the course	☐	☐
I am not interested in any of the research projects on offer	☐	☐
I do not want to lose friends	☐	☐
I already have a BSc/PhD/Other degree	☐	☐
I was anxious about the different style of learning	☐	☐
I thought the course would be difficult	☐	☐
I do not think the degree will help me	☐	☐
Other	☐	☐

Q21 What do you think the benefits of an Intercalated BSc are ? (Tick all that apply)

Gain new skills ☐
Broadens knowledge ☐
Experience in research ☐
Improves learning habits for future studies ☐
It is a challenge ☐
Improves long term career prospects ☐
Will help get a future job ☐
Students get an extra summer holiday ☐
A break from the MBChB course ☐
Study a topic of interest in more depth ☐
Study a topic related to a career in mind ☐
A chance to get a publication ☐
It will be enjoyable ☐
Other ☐

If you have any comments on any aspect of the BSc please write them here (continue overleaf if required). - Thank you for your time
